# Donor-derived TB after kidney transplantation: a case report

**DOI:** 10.1590/2175-8239-JBN-2020-0117

**Published:** 2021-04-09

**Authors:** Luiz Roberto de Sousa Ulisses, Helen Souto Siqueira Cardoso, Inara Creão Costa Alves, Isabela Novais Medeiros, Camilla Garcia de Oliveira, Tiago Martins de Almeida, Fabíola Fernandes dos Santos Castro, Claudia Neto Gonçalves Neves da Silva, Laura Viana de Lima, Renata Pereira Fontoura, Eduardo Resende Sousa e Silva, Pollyana Lopes de Araújo, Gustavo de Sousa Arantes Ferreira

**Affiliations:** 1 Instituto de Cardiologia do Distrito Federal, Brasília, DF, Brasil.; 2 Universidade Católica de Brasília, Brasília, DF, Brasil.

**Keywords:** Tissue Donors, Kidney Transplantation, Tuberculosis, Doadores de Tecidos, Transplante de Rim, Tuberculose

## Abstract

**Introduction:**

Tuberculosis (TB) is a possible serious complication of solid organ transplantation, associated with high mortality and morbidity. Post-transplant TB has varied pathogenesis with many approaches to its prevention, which is the most important way to reduce its incidence. Treatment of TB in organ recipients is challenging because of drug toxicity and interaction with immunosuppressants.

**Case report:**

an 18-year-old woman that underwent kidney transplantation from a deceased donor and was discharged with fair renal function was readmitted at 37th postoperative day with fever. CT showed signs of miliary TB and fluid collection besides graft fistulization through the skin. The patient presented positive BAAR in the drained fluid and Koch's bacillus in the urine. She was treated with a four-drug regimen (rifampicin, isoniazid, pyrazinamide, and etambutol), with great response and preserved graft function. We were informed that the recipient of the contralateral kidney also presented post-transplant TB, implying in a donor-derived origin.

**Conclusion:**

TB is an important differential diagnosis for infectious complications in patients after solid-organ transplantation, especially in endemic regions. Its initial clinical presentation can be unspecific and it should be suspected in the presence of fever or formation of fluid collections. The suspicion of TB is the key to early diagnosis and satisfactory outcomes in post-transplant TB.

## Resumo

**Introdução:**

A tuberculose (TB) é uma possível complicação grave do transplante de órgãos sólidos, associada à alta mortalidade e morbidade. A TB pós-transplante tem patogênese variada com muitas abordagens para sua prevenção, que é a forma mais importante de reduzir sua incidência. O tratamento da TB em receptores de órgãos é um desafio devido à toxicidade dos medicamentos e à interação com imunossupressores.

**Relato de caso:**

uma mulher de 18 anos que foi submetida a transplante renal de um doador falecido e recebeu alta com função renal adequada foi readmitida no 37º dia de pós-operatório com febre. A TC mostrou sinais de TB miliar e coleção de fluidos além de fistulização do enxerto através da pele. A paciente apresentou BAAR positivo no fluido drenado e bacilo de Koch na urina. Ela foi tratada com um esquema de quatro medicamentos (rifampicina, isoniazida, pirazinamida e etambutol), com ótima resposta e função de enxerto preservada. Fomos informados de que o receptor do rim contralateral também apresentou TB pós-transplante, implicando em uma origem derivada do doador.

**Conclusão:**

A TB é um importante diagnóstico diferencial para complicações infecciosas em pacientes após transplante de órgãos sólidos, especialmente em regiões endêmicas. Sua apresentação clínica inicial pode não ser específica e deve ser suspeitada na presença de febre ou formação de coleções de fluidos. A suspeita de TB é a chave para o diagnóstico precoce e desfechos satisfatórios na TB pós-transplante.

## INTRODUCTION

Active tuberculosis (TB) is a possible serious complication of solid organ transplantation, with mortality rates of 6-22% despite treatment 1 ^,^ 2. The treatment of TB in this condition is a serious challenge due to drug-drug interactions and significant drug toxicities, including hepatotoxicity and neuropathy. Post-transplant active TB most commonly is due to reactivation of latent TB in a patient with previous exposure, as a consequence of the immunosuppression. There are protocols described for the identification of this condition in possible candidates, which are especially important in endemic areas^2^.

Also, post-transplant active TB can originate from graft donors. Especially in endemic regions, a risk assessment should be performed in living and deceased possible donors. In a post-transplant TB, donor history of TB can suggest donor-derived TB 1. We present a diagnostic challenge of a kidney transplant patient who developed donor-derived post-transplant TB.

## CASE REPORT

The patient was an 18-year-old woman with Chronic Kidney Disease (CKD) on hemodialysis due to probable kidney dysplasia, with a history of developmental delay due to hydrocephalus (presents ventriculoperitoneal shunt). The preoperative and admission chest radiographs were normal. PPD or IGRA tests were not performed, since they were not part of the current pre-transplant routine. The recipient did not have a medical history suggestive of tuberculosis.

She underwent a kidney transplantation from a deceased donor on 7/10/18. The donor was standard criteria one: a 17-year-old male, whose cause of death was self-extermination (hanging). His creatinine level was 1.55 mg/dL. He was induced with thymoglobulin (panel-reactive antibody, 29%) and maintained with tacrolimus (0.1 mg/kg), mycophenolate sodium, and prednisone. At the time of donation, there was no pulmonary disease report and no chest X-rays were performed. The donor did not have a history that suggested the diagnosis of previous TB.

After the transplant, the patient evolved with immediate graft function and was discharged at the seventh postoperative day with creatinine level at 1.16 mg/dL. She was readmitted at 37th postoperative day with fever and cytomegalovirus positive antigenemia (11 cells) and was treated with ganciclovir for 14 days, but maintained the daily fever. Abdomen ultrasonography and chest X-ray were normal at this time, and sequential cultures (blood and urine) were positive for *Leuconostoc mesenteroides*, being treated with association of ampicillin and gentamicin, although the daily fever persisted.

After 2 weeks of hospitalization without resolution of the clinical condition, a chest computed tomography (CT) was performed, which showed diffuse micronodular disease compatible with miliary TB and abdominal fluid adjacent to the middle third of the transplanted kidney; this fluid fistulized through the skin. At this time, the medical team of the contralateral kidney transplant was contacted who informed that their patient presented a similar condition, being diagnosed with tuberculosis from the donor and had to undergo graft nephrectomy. Also, that team identified in the donor history a hospital admission for complicated pneumonia with pleural effusion one month before his death.

**Figure 1:**
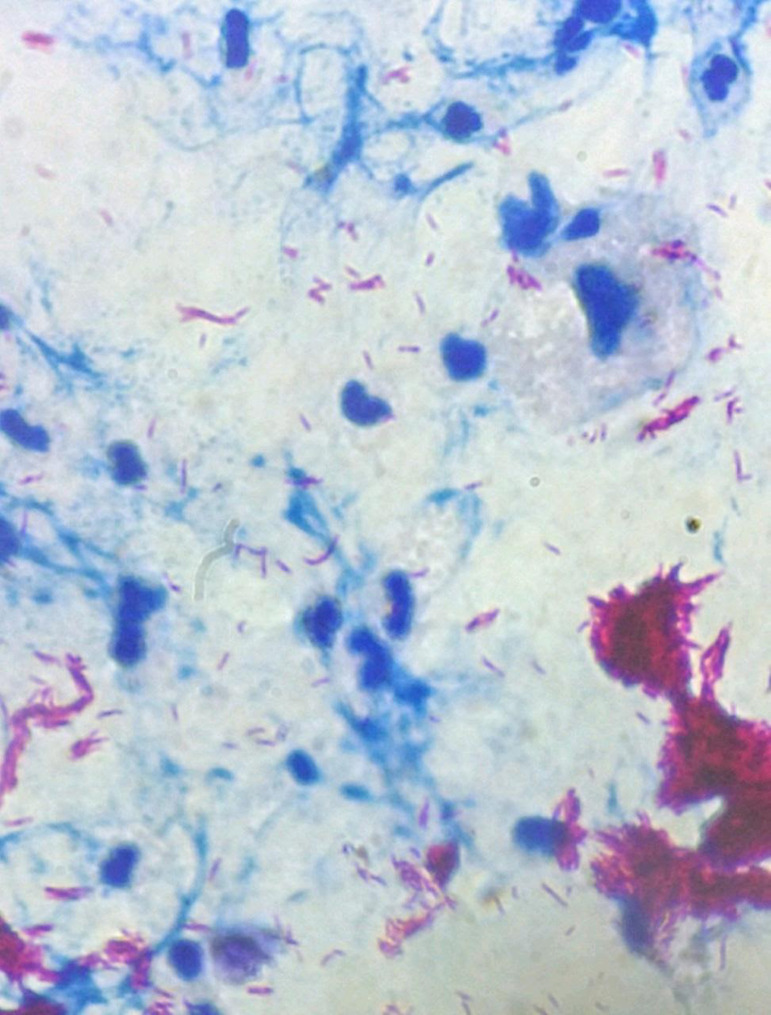
Bacilloscopy by Ziehl Neelsen stain of the fluid drained by the surgical wound, identified as renal fluid.

Our patient presented a positive polymerase chain reaction (PCR) for Koch's bacillus in the urine, and a positive Acid-Alcohol Resistant Bacilli (AARB) test in the fluid drained from the surgical wound, shown in Figure 1. After confirmation of TB diagnosis, the team decided to maintain the graft and started specific treatment with Coxip (rifampicin, isoniazid, pyrazinamide, and ethambutol) along with the suspension of immunosuppressive medication. The Coxip scheme was maintained for 12 months. Prednisone was reintroduced 12 days after the start of TB treatment, and the rest of immunosuppressive medications was resumed after 8 weeks. The creatinine level after the end of TB treatment was 0.85 mg/dL. The patient is presently in outpatient follow-up on tacrolimus, sirolimus, and prednisone and has a creatinine level at 0.76 mg/dL.

## DISCUSSION

Despite the reduction of the TB burden in Latin America in the last two decades, TB remains an important public health issue, with Brazil still being considered an endemic country 1. The incidence of TB is higher on patients after solid organ transplantation and the risk of TB after kidney transplantation is estimated to be 20-74 times greater than the general population 3 ^,^ 4. Reactivation of foci of latent TB infection (LTBI) is considered the main cause of post-transplant TB 1.

The minority of the primary cases of TB after solid organ transplantation are from donor-recipient transmission. Despite the majority of post-transplant donor-derived infections are viral, among the non-viral ones, TB is the most common 5. Most of these cases were associated with deceased donors with active TB, in which the estimated risk of transmission is about 30%. The risk of recipient infection from donors with untreated LTBI is still undetermined. Also, TB bacilli may be present in transplanted organs of donors whose infection may have gone un-diagnosed, as in the presented case 1 ^,^ 6.

The current guidelines mainly recommend the tuberculin skin test (TST) for screening of LTBI in solid organ candidates and recipients and living donors. Nevertheless, this test has some disadvantages: interpretation bias; false-positive results related to previous exposure to others Mycobacteria or BCG vaccination; and cutaneous anergy on patients with CKD 2 ^,^ 4 ^,^ 6 ^,^ 7. IFN-γ release assays (IGRAs) is a new diagnostic method and is probably more sensitive in candidates with chronic renal failure. Although it is more expensive than TST and its negative predictive value is not optimal, it cannot be relied on to exclude the possibility of infection in patients with high risk for LTBI (born in endemic countries, household contacts of TB, contact with homeless shelters) 1.

The screening for TB should start with an investigation of the history of TB in all recipients and donors. In the absence of history, LTBI should be investigated with either TST or IGRA. TST should be preferred in high-risk patients and, in this case, a positive reaction is considered as an induration of 5 mm or greater at 48 to 72 hours. A second TST is needed to evaluate boosted-related skin conversion 7 to 10 days after the first. When positive, active TB must always be excluded. The screening for living donors is the same as for candidates, except that the reference value for TST is 10 mm 1.

Donor-derived TB manifests in the immediate to early post-transplant period and should be considered in patients presenting fever in the first three months after transplantation. The diagnosis can be challenging because the symptoms are usually unspecific. It most commonly presents as a fever that is usually treated as a sign of a non-TB infection. The suspicion of TB must be done when there is no response to empiric antibacterial treatment^7^^,^ 8.

Costa et al. performed a cohort study with 1604 Brazilian patients who underwent kidney transplantation for 20 years and found a post-transplantation TB incidence of 2.1% in a median of 25.5 months from transplant, ranging from 1 to 168 months, in which 11.8% had disseminated TB as in our case. The authors found that TB after kidney transplantation was associated with a higher incidence of acute kidney injury (AKI). Severe AKI and allograft rejection episodes were found to be common events during the TB infection, along with non-recovery of baseline kidney function after infection treatment, depending on the regimen impaired 9.

Another cohort study by Meinerz et al., with 1737 kidney recipients for 12 years, found a TB incidence rate of 5%. Donor-derived TB could not be excluded in 3.3% of the cases. This study found a significant reduction in patient and graft survival. Also, recipients with disseminated disease accounted for 38.8% of the deaths and graft losses. These findings corroborate the importance of prevention and early diagnosis of TB in patients after solid organ transplantation 3.

To prevent donor-derived TB, organs from donors with diagnosed active TB should be discarded and from donors with a history of TB successfully treated for at least 6 months can be transplanted. A history of untreated LTBI without evidence of active infection does not contraindicate the transplant, but preventive therapy should be considered especially for lung transplantation^1^.

The greatest challenge of TB therapeutic approach is the potential toxicity and drug interaction, especially between rifampicin and the immunosuppressive drugs. In general, guidelines recommend a 4-drug regimen, with isoniazid and rifampicin as first choices. There is no consensus over the optimal duration of treatment, but in general, guidelines recommend a 6- to 9-month regimens. Although rifampicin lowers the levels of immunosuppressants, possibly inducing graft dysfunction, it is still indicated even in guidelines that do not recommend its use as first-line drug, restricting the use to disseminated or severe TB. The other drugs mostly used for the treatment of post-transplant active TB are ethambutol and pyrazinamide, such as successfully implemented in our case^1^^,^ 10.

In conclusion, TB is an important differential diagnosis for infectious complications in patients after solid-organ transplantation, especially in endemic regions. Its initial clinical presentation can be unspecific and it should be suspected in the presence of fever or formation of fluid collections. The suspicion of TB is the key to early diagnosis and satisfactory outcomes in post-transplant TB. Our case represents a diagnostic challenge of a rare form of TB pathogeny, in which a serial evaluation was essential for the accurate diagnosis and a well-succeeded therapeutic approach.
